# Unilateral advanced glaucoma in isolated congenital ectropion uveae with ipsilateral ptosis: A pictorial description of five children

**DOI:** 10.22336/rjo.2022.25

**Published:** 2022

**Authors:** Sagarika Snehi, Manpreet Kaur, Ashok Kumar Singh, Faisal Thattarattody, Srishti Raj, Surinder Singh Pandav, Sushmita Kaushik

**Affiliations:** * Advanced Eye Centre, Postgraduate Institute of Medical Education and Research, Chandigarh, India

**Keywords:** congenital ectropion uveae, unilateral ptosis, juvenile glaucoma, Congenital Iris Ectropion Syndrome

## Abstract

**Aim:** To report the cases of five children with unilateral advanced glaucoma in isolated congenital ectropion uveae (CEU) with ipsilateral ptosis and myopia.

**Methods:** This is an ambispective observational case series. After diagnosing one patient with CEU and glaucoma, consecutive patients presenting with unilateral ptosis, congenital iris anomaly, and glaucoma between 2014 to 2020, and had completed a minimum one-year postoperative follow-up, were analyzed.

**Results:** Of the 1421 newly registered pediatric glaucoma patients in the period under review, five children were diagnosed with CEU. All patients presented with gradual painless diminution of vision in the left eye in early adolescence. The left eye of all patients had peculiar clinical features: mild congenital ptosis, high iris insertion, crypt-less smooth iris surface, congenital ectropion uveae, pigments over anterior lens capsule, high myopia, advanced glaucomatous optic disc cupping, and very high intraocular pressure (IOP), which was > 45 mmHg in all cases. The right eye showed signs of angle dysgenesis with mild anterior iris insertion and numerous fine iris processes. Antiglaucoma medications and angle surgery failed to control the IOP, and all children required glaucoma filtration surgery, resulting in reasonable IOP control. Despite the older age, postoperative strict amblyopia treatment resulted in significant improvement in vision.

**Conclusions:** Although ectropion uveae and ptosis have been present since birth, unilaterality, and the asymptomatic nature of the disease led to the late presentation with irreversible damage. Early surgical management and amblyopia therapy are the cornerstones of management.

**Abbreviations:** CEU = Congenital ectropion uvea, CIES = Congenital Iris Ectropion Syndrome, ASD = Anterior segment dysgenesis syndrome, BCVA = Best-corrected visual acuity, IOP = Intraocular pressure

## Introduction

Congenital ectropion uvea (CEU), also termed Congenital Iris Ectropion Syndrome (CIES), is a rare form of anterior segment dysgenesis characterized by the presence of iris pigment epithelium on the anterior surface of the iris [**1**,**2**]. It has been reported to be associated with coloboma [**3**], ptosis [**4**], and systemic diseases like neurofibromatosis type 1 [**5**], Prader-Willi Syndrome [**6**], and facial hemihypertrophy [**1**]. There is scarce literature on CEU, mostly limited to single case reports or small case series.

Glaucoma is a frequent complication of CEU, reported variously in two case series in nine of ten cases [**1**] and seven of eight cases [**2**]. The affected eye may exhibit mild ptosis with good levator function, most likely related to the neural crest origin of the Muller’s muscle [**2**]. We report the occurrence of isolated CEU in five children who presented with advanced glaucoma to a pediatric glaucoma service at our tertiary care institute and presented the typical features of this rare condition in a pictorial description. 

## Methods

The study was an ambispective observational case series. Ethics approval was obtained from the Institute Ethics Committee (No. INT/ 2021/ 1428) of Postgraduate Institute of Medical Education and Research, Chandigarh, India. Parental consent was taken to include the children in the study and use recognizable photographs for academic purposes. 

After diagnosing one child with typical CEU and glaucoma, we wanted to see how common this entity was in our cohort of childhood glaucoma. We screened the clinical records and clinical photographs of children with unilateral CEU who presented to the Paediatric Glaucoma Clinic of the Advanced Eye Centre of Postgraduate Institute of Medical Education and Research, Chandigarh, between July 2014, and June 2020. All children had been previously diagnosed with juvenile glaucoma according to the Childhood Glaucoma Research Network (CGRN) [**7**] criteria, with the presence of any two of the following: IOP > 21 mmHg without medications; optic disc cupping: focal rim thinning, cup-disc ratio > 0.6, cup-disc asymmetry of ≥ 0.2 between the two eyes; corneal findings: Haab striae or diameter ≥ 11 mm in newborn, > 12 mm in child < 1 year, or > 13 mm any age; progressive myopia, myopic shift, or an increase in ocular dimensions out of keeping with normal growth; reproducible visual-field defect consistent with glaucomatous optic neuropathy with no other observable reason for defect.

Children were characterized as suffering from CEU if they fulfilled the following criteria: presence of iris pigment epithelium on the anterior surface of iris with no other characteristic suggestive of any other anterior segment dysgenesis (ASD) syndrome such as Axenfeld Rieger’s anomaly, Peter’s anomaly, or partial aniridia. Neonatal-onset congenital ectropion uveae is a recently described phenotype of newborn glaucoma, which has a bilateral presentation with buphthalmos and cloudy cornea [**8**]. We did not include this entity in the present report. 

Children with a minimum follow-up of one year were included. The data were recorded on standard set proformas, including the following characteristics: intraocular pressure by applanation tonometry, optic disc cupping, presence of ptosis, presence of any systemic conditions, especially neurofibromatosis. The clinical profile, any interventions done, and the outcome after interventions were recorded for all children. 

## Results

After diagnosing CEU in one patient, we could identify four more children from the clinic records in the seven years studied. The presentation and follow-up features of the five children are depicted in **Table 1**. The mean follow-up period was 51.8 ± 22.9 months.

**Table 1:** Clinical features at presentation and last follow-up

	Age (years) Gender	Ptosis **LE	#IOP at presentation (mmHg)		##BCVA at presentation		Axial length (mm)		Management	Follow-up (months)	Last recorded #IOP (mm Hg) without medication		Last recorded ##BCVA	
			*RE	**LE	*RE (unaided)	**LE (Refractive error)	*RE	**LE			*RE	**LE	*RE	**LE
Case 1	7, Male	2 mm	16	48	6/ 6	6/ 24 (-8.5 DS)	24.5	27.2	Goniotomy followed by trabeculectomy	18	14	10	6/ 6	6/ 9 (-8.5 DS)
Case 2	14, Female	2 mm	18	52	6/ 6	6/ 18 (-3.5 DS)	20.5	25.2	Trabeculectomy	57	14	12	6/ 6	6/ 12 (-3.5 DS)
Case 3	16, Male	2 mm	14	50	6/ 6	6/ 24 (-4.0 DS)	23.0	26.2	Trabeculectomy followed by Glaucoma drainage device	82	14	12	6/ 6	6/ 24 (-4.0 DS)
Case 4	17, Female	2 mm	14	48	6/ 6	6/ 24 (-9.5 DS)	23.1	27.9	Trabeculectomy	68	12	14	6/ 6	6/ 24 (-9 DS)
Case 5	14, Female	2 mm	17	54	6/ 6	6/ 18 (-8.0 DS)	22.4	27.1	Trabeculectomy	57	12	14	6/ 6	6/ 12 (-8.25 DS)
Mean ± SD	13.6 ±-3.9	2 mm	15.8 ± 1.78	50.4 ± 2.6		-6.7 DS ± 2.75	22.71 ± 1.45	26.73 ± 1.06		56.4 +-23.79	13.2 +- 1.09	12.4 +- 1.67	6/ 6	-6.6 ± 2.6 DS
*RE: Right eye, **LE: Left eye, #IOP: Intraocular pressure, ##BCVA: Best corrected visual acuity, DS: Diopters sphere														

**Case 1**

**Fig. 1:**
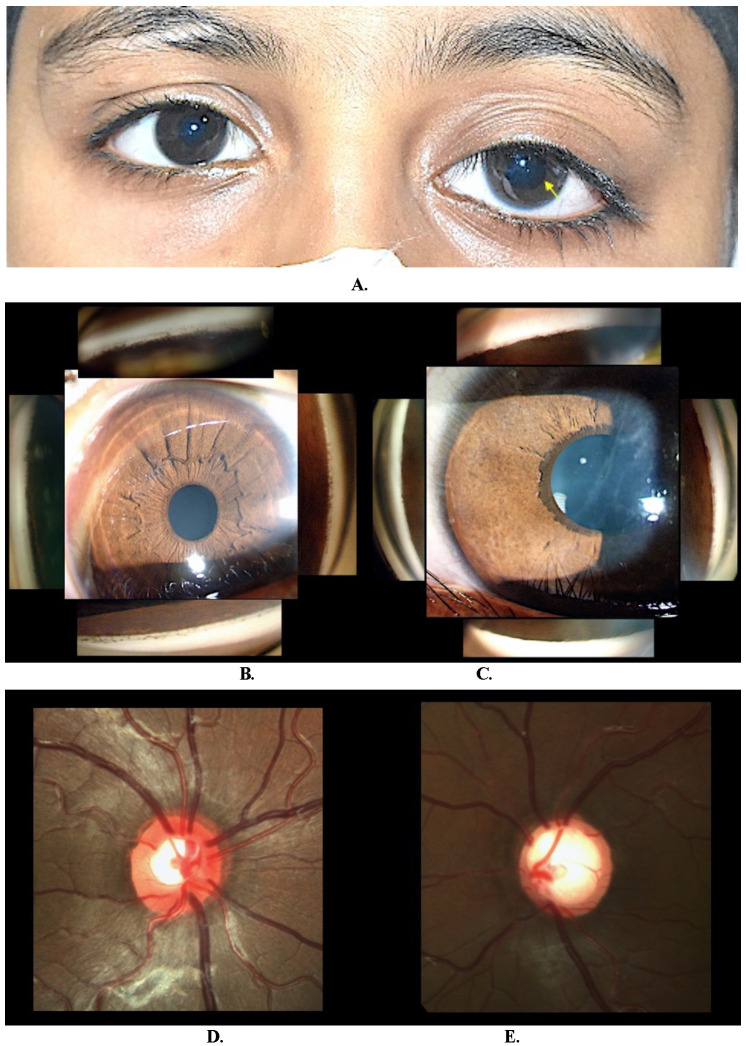
**A.** Clinical image of case 1 showing left eye ptosis, buphthalmos and ectropion uveae (arrow); **B.** The angles of the right eye show numerous iris processes and normal iris pattern; **C.** The left eye showing Haab’s stria, anterior insertion of the iris and a featureless iris surface with ectropion uveae. The pigment on the anterior lens surface should be noted; **D.** The optic nerve was healthy in the right eye; **E.** The left eye shows advanced optic neuropathy

A 7-year-old boy was brought to the hospital when his parents noticed an abnormal pupil with a drooping of the left upper eyelid (**Fig. 1 A**). On examination, the right eye was normal, and the left eye was more prominent, with a best-corrected visual acuity (BCVA) of 6/ 24 with -8.5 Diopters refractive error (**Table 1**). The intraocular pressure (IOP) was 16.0 and 48.0 mmHg in the right and left eye, respectively. The left eye had a smooth, featureless iris surface and ectropion uveae with a high insertion of iris on gonioscopy (**Fig. 1 B**) and pigment deposits on the anterior lens surface (**Fig. 1 C**). The optic nerve was healthy in the right eye (**Fig. 1 D**) and had a near-total optic disc cup in the left eye (**Fig. 1 E**). 

We tried a goniotomy as initial management for raised IOP in the left eye, but it failed to control the IOP, and he required a trabeculectomy with 0.02% mitomycin C applied for 2 minutes. The patient also underwent amblyopia therapy with occlusion of RE. At 18 months of follow-up, the IOP was 10 mmHg without medication, and the BCVA was 6/ 9. Genetic analysis using targeted gene testing revealed no pathogenic variant.

On screening clinical records and photographs retrospectively, of the 1421 patients who presented to the pediatric glaucoma clinic in the six years, between July 2014 and June 2020, we identified four patients with typical features of CIES who had completed a one-year follow-up (3.5%). All children had unilateral involvement in the left eye, mild upper eyelid ptosis, and myopia. Demography and clinical features are depicted in **Table 1**. All patients had in common an anterior iris insertion, concave iris configuration, featureless iris surface with 360o ectropion uveae, anisocoria, pigment deposition on the anterior lens capsule, and advanced glaucomatous optic atrophy. 

**Case 2**

**Fig. 2:**
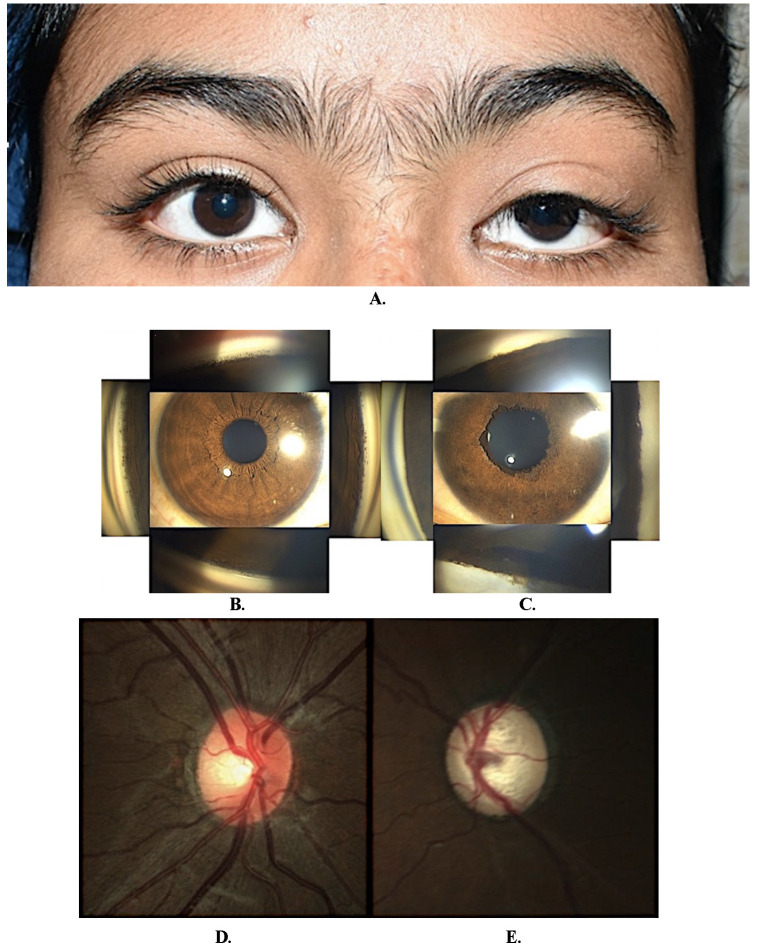
**A.** Ptosis in left eye in case 2; **B.** Slit-lamp examination showing normal iris pattern in the right eye with fine iris processes seen on gonioscopy in all four quadrants; **C.** In the left eye, the iris has a featureless appearance with anisocoria and ectropion uveae. Gonioscopy showing a high iris insertion and concave iris configuration; **D.** Optic disc evaluation showing healthy disc in right eye; **E.** Total glaucomatous optic atrophy in the left eye

The parents of a 14-year-old girl brought her with complaints of drooping of the left upper eyelid noticed since birth (**Fig. 2 A**) and gradually progressive painless diminution of vision in the left eye for two years. On examination (**Table 1**), the right eye was normal (**Fig. 2 B**). The BCVA in the left eye was 6/ 18 with -3.75 Diopter myopia. The IOP was 18 and 52 mmHg in the right and left eye, respectively. A mild 2.0 mm ptosis on the left side with a well-defined lid crease and good levator palpebrae superioris (LPS) muscle action (**Fig. 1 A**) was observed. The left iris had a featureless appearance with 360 degrees ectropion uveae (**Fig. 2 C**). Gonioscopy revealed a high iris insertion at the Schwalbe line (**Fig. 2 C**). Advanced glaucomatous optic neuropathy in the left eye with diffuse peripapillary retinal nerve fiber layer loss was observed, while the right eye disc was normal (**Fig. 2 D, E**).

She underwent a left trabeculectomy with 0.02% mitomycin C (MMC) applied for 2 minutes. At the 5-year follow-up, the IOP was 12 mmHg without drugs, and BCVA was 6/ 12. 

**Case 3**

**Fig. 3:**
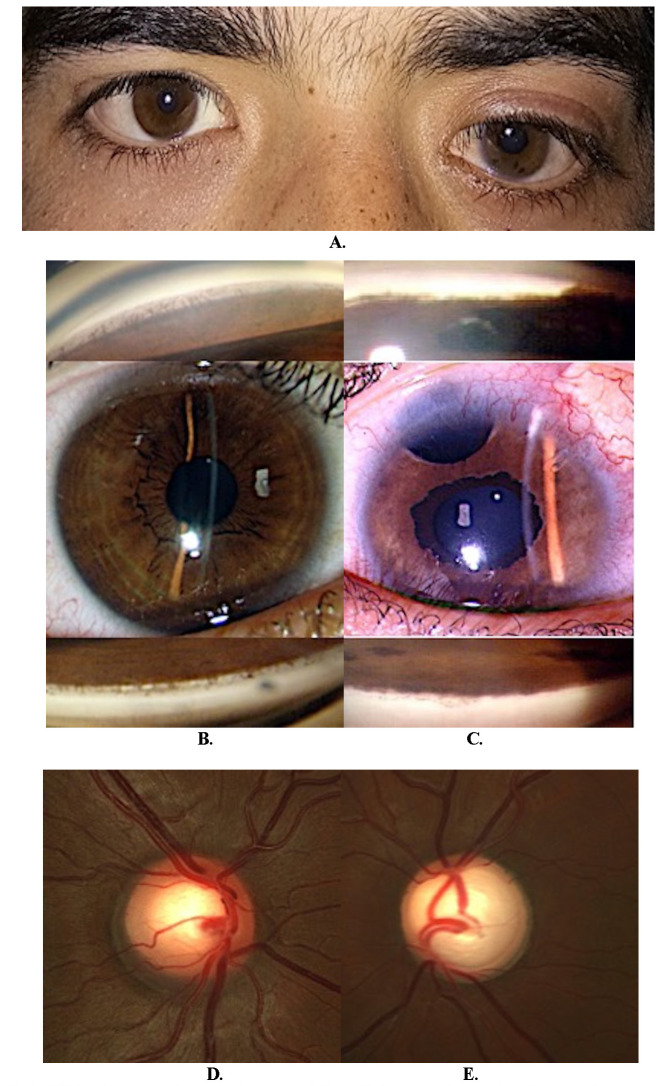
**A.** Clinical image of case 3 showing mild ptosis in the left eye; **B.** Slit-lamp view of the right eye showing concave iris configuration and normal iris pattern; **C.** The left eye shows a smooth featureless anterior iris surface and ectropion uveae involving the pupillary margin; **D, E.** Optic disc assessment showing normal disc in the right eye **(D)** and advanced glaucomatous disc changes in the left eye **(E)**

A 16-year-old boy presented with decreased vision in the LE for three years. On presentation, the BCVA was 6/ 6 and 6/ 24 (with -4.0 DS) in the right and left eye, respectively. The right eye was normal (**Fig. 1 A, B**). The IOP was 14.0- and 50.0-mmHg in the right and left eye. A left-sided mild ptosis that had remained unnoticed (**Fig. 3 A**) was observed. The left eye had a smooth iris surface with patchy pigmentation on the anterior lens surface and a 2-3 mm frill of irregular ectropion uveae (**Fig. 3 C**). The optic discs were large (2.1 mm diameter), with a healthy neuro-retinal rim in the right eye (**Fig. 3 D**) and advanced glaucomatous cupping in the left eye (**Fig. 3 E**).

He underwent trabeculectomy with MMC but, after six months, required a glaucoma drainage device to control the IOP (**Fig. 3 D**). After seven years of follow-up, the IOP is well controlled.

**Case 4**

**Fig. 4:**
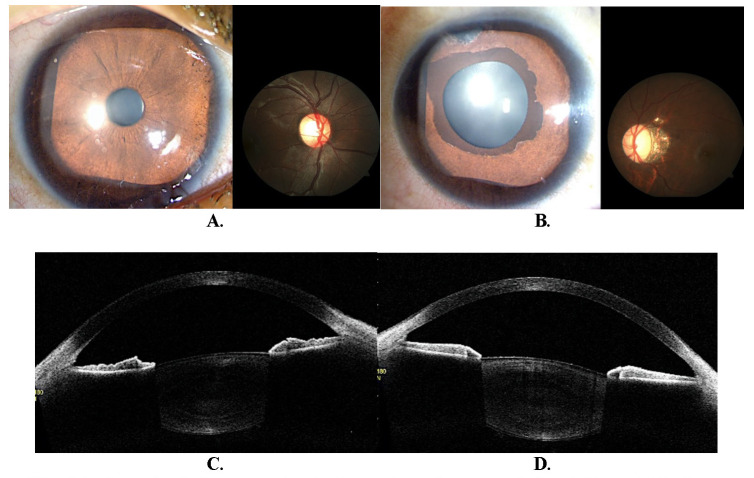
**A.** Normal anterior segment and a large disc and cup seen in the right eye; **B.** Left eye showing ectropion uveae with a glassy, smooth, crypt less iris surface and oblique optic disc with temporal peripapillary atrophy and significant cupping; **C.** Anterior segment OCT (ASOCT) image of the right eye showing open angles, concave iris configuration and normal iris pattern; **D.** The ASOCT in the left eye showing an anterior insertion of iris. The hyperreflective particles should be observed on anterior lens capsule, which may be due to pigment deposition

A 17-year-old girl presented with diminution of vision and drooping of the left upper eyelid for the past ten years. She was diagnosed with glaucoma in the left eye and underwent left eye trabeculectomy with MMC elsewhere seven years before. The right eye was normal (**Fig. 4 A**). The BCVA was at presentation was 6/ 6 in the RE and 6/ 24 in the LE (with -9.5 DS), and the IOP was 48.0 mmHg in the left eye. A 2.0 mm ptosis was noticed in the LE with good LPS function (13 mm). The left eye’s iris showed a smooth surface, marked ectropion uveae encompassing ¼ of the visible iris surface, terminating in a sharply demarcated border (**Fig. 4 B**). The swept-source anterior segment OCT (Tomey-Cassia, SS-1000®) also showed obscured anterior chamber angle with high iris insertion and hyper-reflective pigments on the anterior lens capsule (**Fig. 1 D**).

The fundus evaluation revealed oblique disc insertion in the LE with temporal peripapillary atrophy, the cup-disc ratio was 0.8, and a profound loss of the surrounding RNFL (**Fig. 4 B**) was observed.

**Case 5**

**Fig. 5:**
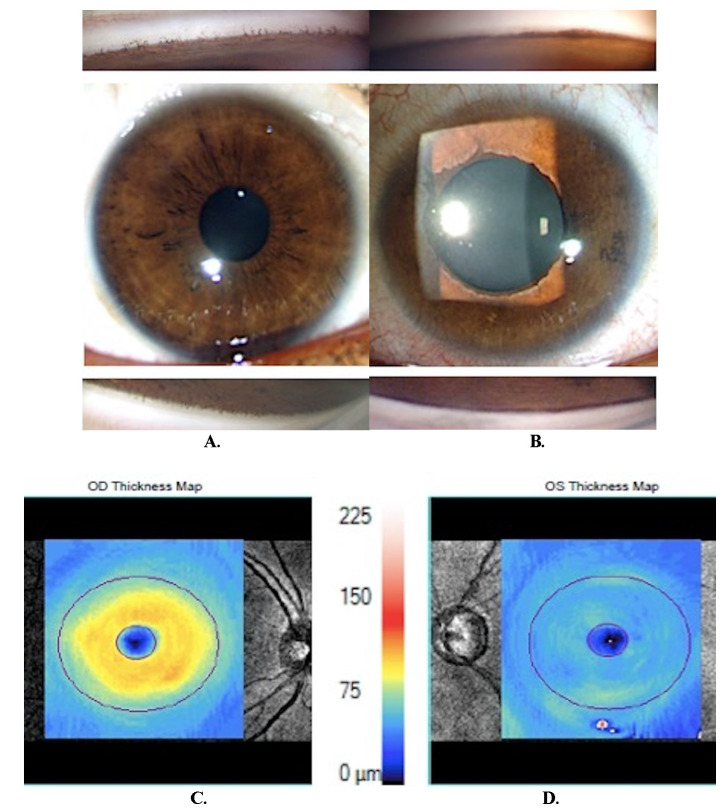
**A.** Right eye showing normal anterior segment and fine iris processes on gonioscopy; **B.** Left eye showing an anteriorly inserted iris on gonioscopy and featureless iris surface with ectropion uveae involving entire pupillary margin. The marked anisocoria should be noted; **C, D.** Macular ganglion cells assessment showing normal thickness in right eye **(C)** and near-total loss of ganglions in the left eye **(D)**

A 14-year-old girl presented with decreased vision and raised IOP in the left eye with unilateral myopia (-8.0 D). The right eye was normal (**Fig. 5 A, C**). The gonioscopy and clinical features of the left eye were typical of CEU, with anteriorly inserted iris root, and a smooth iris surface with ectropion uveae (**Fig. 5 B**). She had advanced glaucomatous optic neuropathy in the LE and near-total loss of peripapillary RNFL and macular ganglion cells (**Fig. 5 D**). She underwent trabeculectomy with MMC, and at the five-year follow-up, the BCVA was 6/ 12 and IOP was 14 mmHg.

Since ectropion uveae is frequently associated with systemic disorders, the patients were recalled and examined to detect any signs of probably associated diseases. The skin examination was normal, and no café-au-lait spot or any other sign of neurofibromatosis was observed. The dental examination was also normal. Their mental and intellectual abilities were age-appropriate. 

## Discussion

CEU is a rare anterior segment dysgenesis. It has been reported from infancy to early adulthood [**9**-**12**]. The underlying mechanism of glaucoma has been postulated to be a delayed neural crest developmental arrest leading to a failure of regression of embryological remnants and lack of migration of iris tissue during anterior segment development [**13**].

Wicherkiewicz [**14**] first described congenital ectropion in 1891 and Spiro [**15**] in 1896. The underlying angle dysgenesis results in the inevitable occurrence of glaucoma [**1**,**2**,**4**,**10**]. When associated with neurofibromatosis, the pathogenesis of glaucoma has been described to be secondary to endothelialization of the anterior chamber angle, sometimes resulting in angle-closure [**5**]. The diagnosis of isolated CEU is often missed, which may result in unrecognized glaucoma detected only in the advanced stage. 

In six years, we found CEU in 5 of 1421 (3.5%) newly registered children with glaucoma in our clinic. None of our children had any systemic abnormalities to suggest neurofibromatosis. Isolated CEU syndrome is usually unilateral and tends to be non-progressive [**16**]. All five children were remarkably identical in their presentation with classical features of Isolated CEU, typically, a featureless iris, CEU, anterior iris insertion on gonioscopy, angle dysgenesis, and advanced glaucoma. One peculiar feature was the pigmentation of the anterior lens surface of all children, which may be a marker of the developmental arrest of the primordial endothelium and may represent immature remnants of embryological tissue.

All five children had significant axial myopia, indicating that the raised IOP may have been present prior to 3 years, resulting in a buphthalmos. Axial myopia itself may be an association of isolated CEU unrelated to the IOP. Since all children were between 7-15 years of age, this cannot be clearly established. The affected eye in all children exhibited mild ptosis with good levator function, probably related to the neural crest origin of the Muller’s muscle [**2**]. Ritch et al. reported ptosis in one of their eight cases [**2**], and Dowling et al. reported ptosis in six of ten cases with CEU [**1**]. 

All children had very high IOP and advanced glaucoma at the presentation. All our patients presented to our clinic in their early teens except for case 1, whom we detected at seven years. Interestingly, the parents noticed the mild ptosis as “one eye being smaller” early in life, and all pediatricians and ophthalmologists reassured them that since it did not cover the pupil, there was no urgency for correction, and they could wait until the child was older. The degree of the glaucomatous optic disc damage and the level of the IOP indicated elevated pressures in the eye for quite some time before the presentation. 

The IOP did not respond to angle surgery, which indicated that it was not an isolated angle dysgenesis, though it is also known that older children frequently respond poorly to angle surgery [**17**]. One patient (Patient 3) required a glaucoma drainage device after the trabeculectomy also failed. Previous reports also indicate that glaucoma is often intractable in these children. Dowling et al. [**1**] reported performing six goniotomies in three patients with glaucoma associated with CEU that did not control IOP, after which they finally required trabeculectomy. Trabeculectomy appears to be largely successful in this condition [**18**,**19**]. Lim et al. [**18**] described three children with isolated CEU, who could successfully control glaucoma over 5-15 years after trabeculectomy with MMC. 

## Conclusion

It is of paramount importance to keep in mind that the coexistence of congenital ectropion with mild ptosis could be harboring severe glaucoma in a young child. In addition, it is crucial to identify this readily recognized condition that requires prompt and aggressive therapy to prevent irreversible blindness in the eyes of these young children. We hope that the pictorial depiction of five cases in this report will help the early identification and prompt treatment.

**Conflict of Interest Statement**

The authors state no conflict of interest. 

**Informed Consent and Human and Animal Rights statement**

Informed consent has been obtained from the parents of the patients included in the study.

**Authorization for the use of human subjects**

Ethical approval: The research related to human use complies with all the relevant national regulations, institutional policies, it is in accordance with the tenets of the Helsinki Declaration and has been approved by the Institute Ethics Committee (No. INT/ 2021/ 1428) of Postgraduate Institute of Medical Education and Research, Chandigarh, India. 

**Acknowledgements**

None. 

**Sources of Funding**

None. 

**Disclosures**

None. 
